# Comparative Efficacy of Interleukin Inhibitors and JAK Inhibitors in Moderate to Severe Plaque Psoriasis

**DOI:** 10.7759/cureus.114260

**Published:** 2026-08-10

**Authors:** Alexandra Daily, London Danchulis, Kiarra Krulikowski, Brittany Shectman, Suzanne I Riskin

**Affiliations:** 1 Department of Foundational Sciences, Nova Southeastern University Dr. Kiran C. Patel College of Osteopathic Medicine, Clearwater, USA; 2 Department of Foundational Sciences, Nova Southeastern University Dr. Kiran C. Patel College of Osteopathic Medicine, Fort Lauderdale, USA

**Keywords:** autoimmune, bimekizumab, deucravacitinib, interleukin inhibitor, jak inhibitor, medication, oral, plaque psoriasis, psoriasis, risankizumab

## Abstract

Plaque psoriasis is a chronic autoimmune skin condition characterized by erythematous, scaly plaques that commonly affect the scalp, trunk, and extensor surfaces. As the most prevalent form of psoriasis, it results from immune system dysregulation, particularly involving cytokines. Cytokines such as interleukin-17 (IL-17) and interleukin-23 (IL-23), as well as downstream Janus kinase (JAK)/tyrosine kinase 2 (TYK2) signal pathways, lead to accelerated skin cell turnover. While an expanding array of treatment options exists, including topical agents, conventional oral medications, and targeted biologics, gaps persist in optimizing long-term management, such as improving access to medications and balancing efficacy with patient preference. Effective, practical, and acceptable long-term treatment options are needed. Injectable interleukin inhibitors, such as risankizumab (Skyrizi^©^) and bimekizumab (Bimzelx^©^), have demonstrated significant efficacy in treating moderate to severe plaque psoriasis. However, the recent FDA approval of deucravacitinib (Sotyktu^©^), an oral TYK2-selective JAK inhibitor, presents a promising alternative that may represent an important oral alternative for selected patients, particularly for patients who are needle-averse, have limited access to biologic infusions, or prefer oral therapies. This structured narrative review evaluates the current evidence surrounding leading IL-17 and IL-23 inhibitors and evaluates the potential role of JAK/TYK2 inhibition in addressing gaps in care. By comparing efficacy, safety profiles, and practical considerations across these agents, this review highlights the need for further longitudinal studies and real-world evidence to guide individualized treatment planning and broaden access to patient-centered care.

## Introduction and background

The development of plaque psoriasis is both immunological and dermatological in nature. Central to its pathogenesis are cytokine-mediated pathways, particularly the interleukin-23/T helper cell 17 (IL-23/Th17) axis. Skin resident immune cells, including dendritic cells, release IL-23, which promotes differentiation and activation of Th17 cells. These cells produce pro-inflammatory cytokines, including IL-17, which drive inflammatory responses within the skin. This cytokine activity creates a positive feedback loop that amplifies inflammatory mediator production and promotes keratinocyte proliferation. The result is epidermal hyperplasia and the formation of the thickened plaques characteristic of psoriasis [1]. 

Despite advances in understanding disease mechanisms, several challenges remain in the management of plaque psoriasis. These include identifying key regulators of plaque formation, establishing safe and effective treatment strategies, and achieving consistent long-term disease control. Effective treatment selection is essential for improving patient outcomes, reducing disease burden, and preventing systemic complications. 

Traditional treatment approaches include over-the-counter preparations, prescription topical therapies, oral systemic agents, phototherapy, and biologic therapies. Topical treatments are often insufficient for moderate to severe disease and may not address systemic involvement. Concerns also remain regarding the long-term safety and durability of corticosteroid use [2]. Among oral systemic agents, phosphodiesterase-4 (PDE-4) inhibitor apremilast has demonstrated lower plaque clearance rates in comparative trials evaluating deucravacitinib and is commonly associated with gastrointestinal adverse effects and weight loss [3]. Biologic therapies have improved disease control for many patients; however, they require parenteral administration and are associated with an increased risk of infection, along with variability in long-term therapeutic response [4]. 

Risankizumab (Skyrizi®) and bimekizumab (Bimzelx®) target the IL-23 and IL-17 cytokines, respectively. IL-23, produced primarily by dendritic cells, stimulates IL-17 secretion from differentiated Th17 cells. IL-17 plays a central role in recruiting macrophages and neutrophils, fueling inflammation and driving epidermal changes through keratinocyte activation and hyperplasia. By inhibiting IL-23 and IL-17, these agents reduce inflammatory signaling and limit keratinocyte overproduction, ultimately decreasing plaque formation [5]. 

Janus kinase (JAK) inhibition has emerged as an alternative therapeutic approach for plaque psoriasis through modulation of intracellular cytokine signaling via the JAK-signal transducer and activator of transcription (JAK-STAT) pathway. These agents influence both innate and adaptive immune responses. Deucravacitinib (Sotyktu®) is a highly selective tyrosine kinase 2 (TYK2) inhibitor that reduces downstream Th17 activity and inflammatory cytokine production. Its selective mechanism allows for targeted modulation of immune signaling while minimizing off-target effects associated with broader JAK inhibition [6]. 

Plaque psoriasis is a prevalent condition worldwide and is associated with a substantial disease burden and multiple comorbidities. Patients receiving nonbiologic systemic therapies often report greater perceived disease burden and less improvement in Dermatology Life Quality Index (DLQI) scores compared with those receiving biologic therapies, even when objective measures such as body surface area involvement appear limited [7]. This discrepancy highlights the importance of incorporating patient-reported outcomes (PROs) alongside clinical measures when assessing disease impact and guiding treatment decisions. Additionally, the overlap between inflammatory pathways in psoriasis and cardiovascular disease underscores the need to consider both dermatologic and systemic health in therapeutic selection [8]. These factors highlight the continued need for optimized treatment strategies. The objective of this paper is to highlight emerging therapies, including selective TYK2 inhibitors, which may help address existing therapeutic gaps by offering alternative mechanisms of action and improving treatment accessibility and adherence. 

This article was accepted for presentation as a meeting abstract at the 2025 American Medical Association (AMA) Interim Meeting Poster Showcase on November 14, 2025.

## Review

Methods

A comprehensive literature search was conducted to identify studies evaluating the mechanism of action, efficacy, and safety of IL-17/IL-23 inhibitors risankizumab, bimekizumab, and TYK2 inhibitor deucravacitinib, and other biologic or small-molecule agents in relation to comparison with deucravacitinib in the treatment of moderate to severe plaque psoriasis. Searches were performed across PubMed, Embase, and Google Scholar. The literature search was conducted on the following dates: PubMed (January 20-29, 2025), Embase (March 9, 2025), and Google Scholar (March 9, 2025).

Search terms included a combination of 'psoriasis,' 'plaque psoriasis,' 'biologic therapy,' 'TYK2 inhibitor,' 'deucravacitinib,' 'IL-17,' 'IL-23,' 'risankizumab,' 'bimekizumab,' and 'immune modulation.' Boolean operators were used to combine terms, resulting in the following core strategy: ('psoriasis' OR 'plaque psoriasis') AND ('biologic therapy' OR 'targeted immune therapy' OR 'TYK2 inhibitor' OR 'deucravacitinib' OR 'bimekizumab' OR 'risankizumab' OR 'IL-17' OR 'IL-23' OR 'JAK inhibitor'). Database-specific syntax adjustments were made for Embase and Google Scholar, and filters were applied to include only English-language human studies published between 2010 and 2025. Representative search strings are provided in the Appendices. Reference lists of relevant reviews were also hand-searched to identify additional eligible studies.

In total, 4,120 records were identified across all databases. After removal of duplicate records (n = 845) and exclusion of articles published outside the predefined date range or lacking full-text availability (n = 760), 2,515 records remained for title and abstract screening. Of these, 2,380 records were excluded at the title and abstract screening stage because they did not meet predefined relevance criteria, including studies focused on non-plaque psoriasis populations, unrelated disease states, non-systemic therapies, or interventions outside the scope of risankizumab, bimekizumab, or deucravacitinib. The remaining 135 full-text articles were assessed for eligibility, of which 114 were excluded based on predefined exclusion criteria.

Studies were included if they met the following criteria: (1) involved adult patients (≥18 years) with moderate to severe plaque psoriasis; (2) evaluated risankizumab, bimekizumab, or deucravacitinib; (3) reported clinical efficacy outcomes (e.g., Psoriasis Area and Severity Index (PASI) scores), safety outcomes, or PROs; and (4) were randomized controlled trials, cohort studies, relevant observational studies, cross-sectional studies, quasi-experimental studies, case-control studies, and review articles that provided relevant clinical, mechanistic, or safety data.

Studies were excluded if they (1) focused on non-plaque psoriasis subtypes; (2) evaluated non-systemic therapies only; (3) did not report clinical outcomes; or (4) were editorials, commentaries, or non-peer-reviewed sources. Studies involving other immune-mediated diseases were included selectively to provide mechanistic or safety context when psoriasis-specific data were limited and were not used to support disease-specific efficacy conclusions.

**Figure 1:**
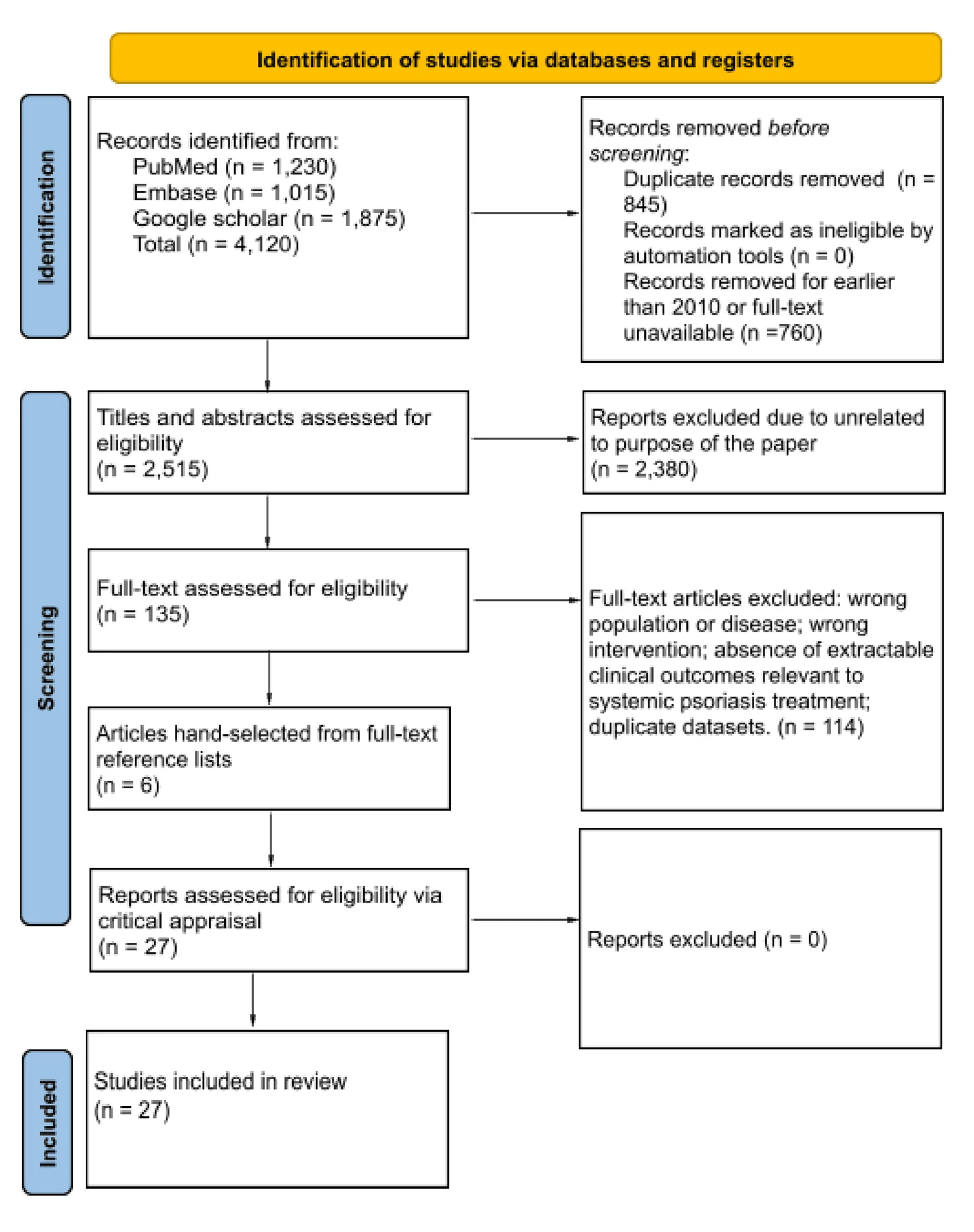
Preferred Reporting Items for Systematic Reviews and Meta-Analyses (PRISMA) 2020 flow diagram for new systematic reviews, which included searches of databases and registers only.

Following database searching, all records were screened for eligibility. After removal of duplicate records and studies outside the predefined date range or lacking full-text availability, titles and abstracts were assessed for relevance. Title and abstract screening were performed independently by four reviewers (AD, LD, KK, BS). Studies deemed potentially eligible underwent full-text review, which was also conducted independently by the same four reviewers (AD, LD, KK, BS). Discrepancies between reviewers were resolved through discussion and consensus. Reasons for exclusion at the full-text stage were documented. Study selection was conducted in accordance with Preferred Reporting Items for Systematic Reviews and Meta-Analyses (PRISMA) guidelines, and the selection process is summarized in the PRISMA flow diagram (Figure 1).

Data extraction was performed by designated reviewers using a standardized extraction framework. Extracted variables included study design, population characteristics, interventions, comparator groups, and outcomes (including PASI response rates, safety data, and PROs). Extracted data were reviewed and verified by additional co-authors to ensure accuracy and consistency prior to analysis.

Included studies were categorized into two groups for analysis. A predefined subset of studies reporting extractable and comparable clinical efficacy and/or safety outcomes (n = 12) was included in the structured comparative analysis presented in the Results section. These studies were selected based on the availability of clearly reported outcome measures (e.g., PASI scores, safety endpoints) and sufficient methodological consistency to allow meaningful comparison across therapies.

The remaining studies (n = 27 total included) were incorporated into the broader narrative synthesis and were used to provide contextual, mechanistic, or supportive clinical insights discussed primarily in the Discussion section. These studies contributed to interpretation of treatment mechanisms, safety considerations, and real-world applicability but were not used for direct comparative outcome analysis.

The methodological quality of included studies was assessed using Joanna Briggs Institute (JBI) critical appraisal tools appropriate to study design, including tools for randomized controlled trials, cohort studies, cross-sectional studies, and case-control studies. Risk of bias assessments were summarized and incorporated into the interpretation of findings.

A hand search through the included articles’ reference lists was performed for relevant background information, which produced six additional articles to be included in the JBI critical appraisal. The JBI critical appraisal results, as well as the population, intervention, comparison, outcomes (PICO) framework for the 27 articles, are summarized in Tables 1-2. All studies meeting inclusion criteria (n = 27) were retained in the systematic review and contributed to the overall synthesis, including background context, mechanistic insights, and therapeutic considerations. Data extracted from included studies encompassed study design, population characteristics, interventions, outcomes, and key findings. A comprehensive database search log is included in the Appendices. 

**Table 1:** Joanna Briggs Institute (JBI) critical appraisal summary of included studies.

Study (Author, Year)	Study Design	JBI Appraisal Tool	Overall Risk of Bias
Hawkes et al., 2017 [1]	Review	JBI Review Checklist	Moderate
Lebwohl et al., 2021 [2]	Review	JBI Review Checklist	Moderate
Strober et al., 2023 [3]	Randomized controlled trial	JBI RCT Checklist	Low
Quartuccio et al., 2019 [4]	Case-control	JBI Case-Control Checklist	Moderate
Tokuyama et al., 2020 [5]	Review	JBI Review Checklist	Moderate
Catlett et al., 2023 [6]	Quasi-experimental	JBI Quasi-Experimental Checklist	Low
Robinson et al., 2023 [7]	Cross-sectional	JBI Cross-Sectional Checklist	Moderate
Reali et al., 2024 [8]	Review	JBI Review Checklist	Moderate
Oliver et al., 2022 [9]	Randomized controlled trial	JBI RCT Checklist	Low
Blauvelt et al., 2020 [10]	Randomized controlled trial	JBI RCT Checklist	Low
Ramakrishna et al., 2024 [11]	Review	JBI Review Checklist	Moderate
Roskoski, 2023 [12]	Review	JBI Review Checklist	Moderate
Papp et al., 2023 [13]	Randomized controlled trial	JBI RCT Checklist	Moderate
Gordon et al., 2021 [14]	Randomized controlled trial	JBI RCT Checklist	Low
Armstrong et al., 2024 [15]	Cross-sectional	JBI Cross-Sectional Checklist	Moderate
Strober et al., 2024 [16]	Cohort	JBI Cohort Checklist	Moderate
Korman et al., 2024 [17]	Cohort (post hoc randomized controlled trial analysis)	JBI Cohort Checklist	Moderate
Armstrong et al., 2025 [18]	Cohort	JBI Cohort Checklist	Moderate
Loftus et al., 2023 [19]	Cohort	JBI Cohort Checklist	Moderate
Zeidan et al., 2022 [20]	Cross-sectional	JBI Cross-Sectional Checklist	Moderate
Armstrong et al., 2024 [21]	Cohort/randomized controlled trial extension	JBI Cohort Checklist	Moderate
Kuang et al., 2024 [22]	Cross-sectional	JBI Cross-Sectional Checklist	Moderate
Gordon et al., 2018 [23]	Randomized controlled trial	JBI RCT Checklist	Low
Armstrong et al., 2024 [24]	Quasi-experimental	JBI Quasi-Experimental Checklist	Moderate
Fleischmann et al., 2024 [25]	Cohort	JBI Cohort Checklist	Moderate
Silverberg et al., 2024 [26]	Randomized controlled trial	JBI RCT Checklist	Low
Armstrong et al., 2023 [27]	Quasi-experimental	JBI Quasi-Experimental Checklist	Moderate

**Table 2:** Population, intervention, comparison, and outcome summary. DLQI = Dermatology Life Quality Index, EASI = Eczema Area and Severity Index, FDA = Food and Drug Administration, JAK = Janus kinase, PASI = Psoriasis Area and Severity Index, PK/PD = pharmacokinetics/pharmacodynamics, sPGA = Static Physician's Global Assessment

Population	Intervention	Comparison	Outcomes
Age 18-80 years with plaque psoriasis	Risankizumab, bimekizumab, deucravacitinib	Placebo; FDA-approved biologic treatments for plaque psoriasis; FDA-approved oral treatments for plaque psoriasis; JAK inhibitors	Efficacy, safety, PASI, EASI, preferences, PK/PD, DLQI, sPGA

A predefined subset of studies (n = 12) that reported extractable clinical efficacy and/or safety outcomes for risankizumab, bimekizumab, and/or deucravacitinib in plaque psoriasis were included in the comparative efficacy analysis presented in the results section. Studies not included in the comparative analysis were retained to support contextual interpretation and methodological completeness. All narrative synthesis, interpretation, and contextual discussion presented in this manuscript were written by the authors. 

A structured narrative synthesis approach was used to analyze and compare study findings. Studies were grouped by therapeutic class (IL-17 inhibitors, IL-23 inhibitors, and TYK2 inhibitors) and compared based on efficacy outcomes (e.g., PASI response rates), safety profiles, and PROs. Due to heterogeneity in study design, populations, and outcome reporting, a formal quantitative synthesis was not performed.

No formal quantitative synthesis (e.g., meta-analysis or meta-regression) was conducted. Reported statistical outcomes, including effect estimates, confidence intervals, and p-values, were extracted from individual studies as presented and were not pooled. Comparative conclusions are therefore based on qualitative assessment of reported results rather than statistical aggregation.

Review

Mechanism of Action of Bimekizumab

IL-17 is a cytokine produced by Th17 cells that plays a critical role in immune defense, particularly in response to infection, through both innate and adaptive pathways. Two main isoforms, IL-17A and IL-17F, form homodimers or heterodimers, which initiate inflammatory signaling through the IL-17 receptor complex (IL-17RA/RC). This signaling cascade promotes recruitment of pro-inflammatory cells, including neutrophils and macrophages, and activates fibroblasts and epithelial cells. Neutrophils serve as key mediators of the inflammatory process, while macrophages can polarize toward an M1 phenotype, producing primary inflammatory cytokines such as tumor necrosis factor-alpha (TNF-α), IL-1, and IL-6 [9]. Fibroblast and keratinocyte activation further drives chemokine release, which attracts additional neutrophils, amplifying inflammation and contributing to the development of psoriatic lesions. The IL-17F isoform is particularly important in the inflammatory component of psoriatic plaques. While some monoclonal antibodies, such as secukinumab, selectively inhibit IL-17A, bimekizumab targets both IL-17A and IL-17F, which may lead to a more pronounced reduction in plaque burden. For this reason, bimekizumab was selected as the most efficacious IL-17 inhibitor for comparison in this review [9].

Mechanism of Action of Risankizumab

IL-23 is another key inflammatory cytokine that plays a central role in the pathogenesis of plaque psoriasis. It is essential for the differentiation, proliferation, and maintenance of Th17 cells, which play a large role in driving chronic inflammation and maintenance of plaque presence in psoriatic lesions. The IL-23/Th17 axis is actively involved in the release of downstream cytokines, including IL-17, which contribute to keratinocyte hyperproliferation and the formation of psoriatic plaques. Therapeutic inhibition of IL-23 has focused on targeting its unique p19 subunit, which is distinct from the p40 subunit shared with IL-12. This selective blockade allows for effective suppression of IL-23 signaling without interfering with IL-12-mediated immune functions. Risankizumab, a humanized monoclonal antibody with high affinity for the IL-23 p19 subunit, has demonstrated substantial reductions in psoriatic disease activity and symptom severity in clinical trials [10].

Mechanism of Action of Deucravacitinib

**Figure 2:**
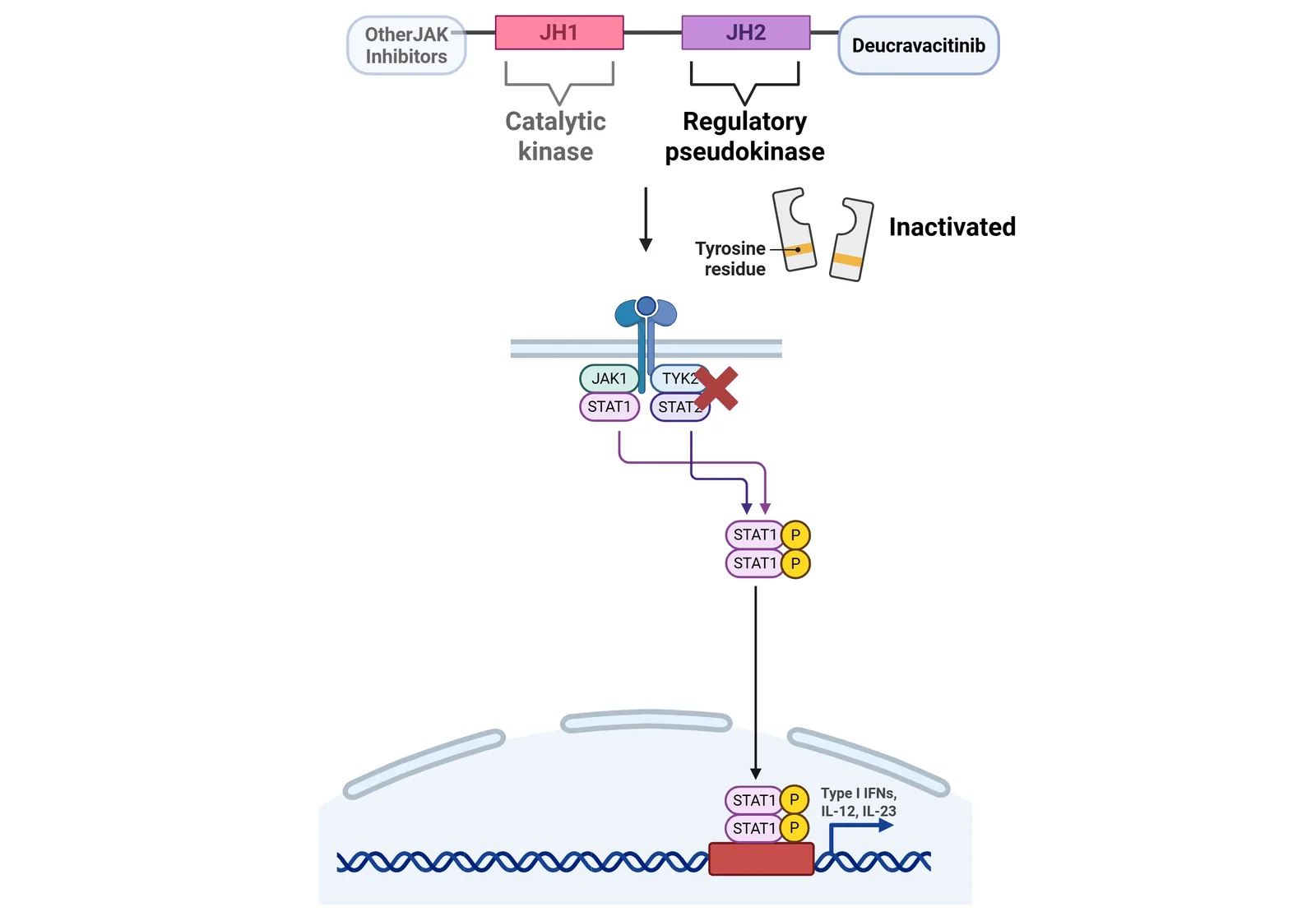
Mechanism of action of deucravacitinib. Created using BioRender (https://BioRender.com/d8cgevd) (Riskin, 2026).

As shown in Figure 2 below, the JAK-signal transducer and activator of transcription (JAK-STAT) pathway is a crucial signaling mechanism in immune function, inflammation, metabolism, hematopoiesis, and cell proliferation. The therapeutic potential of the JAK-STAT pathway is rapidly evolving, expanding the boundaries of treatment strategies for autoimmune diseases, cancer, and inflammatory disorders [11]. Dysregulated JAK-STAT signaling contributes to excessive immune activation, making it a prime target for therapies in plaque psoriasis. When these regulatory mechanisms fail or become dysregulated, JAK inhibitors are employed as targeted therapeutic agents, designed to modulate and restore proper function within the JAK-STAT pathway. JAK inhibitors work by selectively binding to and blocking the activity of one or more JAK molecules. By inhibiting the kinase activity of JAKs, these inhibitors prevent the phosphorylation of tyrosine residues on cytokine receptors, which are necessary to serve as docking sites for STAT proteins. Without phosphorylation, STAT proteins will not bind to the receptor, preventing their activation, dimerization, and nuclear translocation [12]. As a result, the downstream transcriptional activation of pro-inflammatory genes is suppressed, effectively halting the immune cascade. The selective inhibition of JAK molecules allows for more targeted modulation of the immune response, which is essential for devising precise treatment strategies tailored to specific diseases [12].

**Figure 3:**
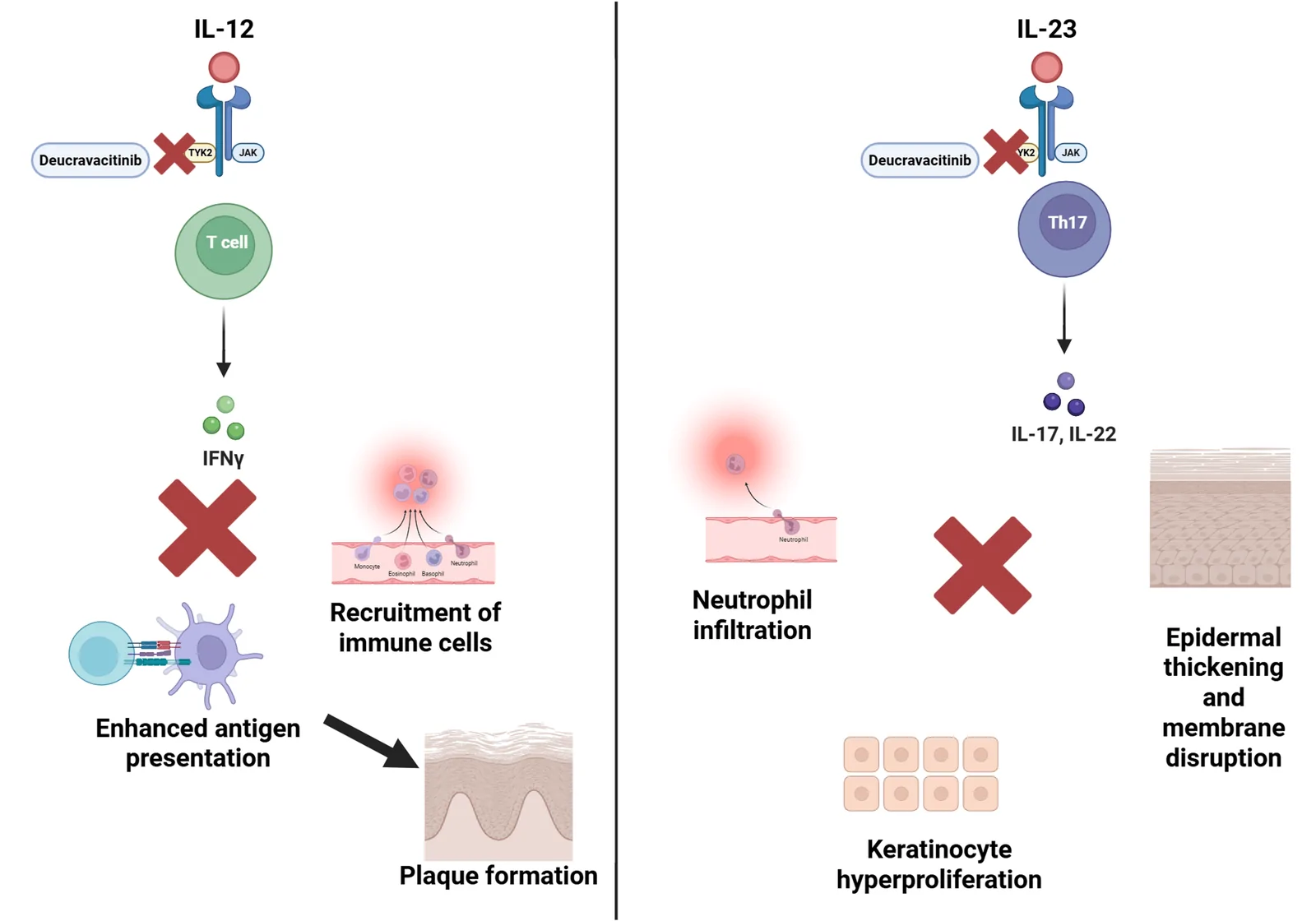
Effects of deucravacitinib inhibition on cytokine function. Created using BioRender (https://BioRender.com/gheq0i8) (Riskin, 2026).

Deucravacitinib is an oral, highly selective TYK2 inhibitor that has emerged as a notable treatment option for plaque psoriasis. It works through an allosteric inhibition mechanism that suppresses inflammatory signaling while limiting off-target effects on JAK1, JAK2, and JAK3. In contrast to traditional JAK inhibitors, which broadly inhibit multiple members of the JAK family, deucravacitinib was engineered to selectively target TYK2 with high precision [11]. TYK2 plays a critical role in regulating immune responses by transmitting signals from various cytokines, including type 1 interferons (IFN-alpha (IFNα)/beta), IL-12, and IL-23. These cytokines are essential in both innate and adaptive immune pathways, influencing Th1 and Th17 cell differentiation as well as a range of inflammatory responses [11]. Deucravacitinib’s selectivity is achieved through its unique binding to the regulatory JH2 (pseudokinase) domain of TYK2, rather than the active JH1 kinase domain. This binding stabilizes TYK2 in an inactive conformation, preventing phosphorylation of downstream STAT proteins while preserving critical immune functions regulated by other JAK family members. Through this targeted action, deucravacitinib effectively disrupts the IL-12 and IL-23 signaling cascade in plaque psoriasis, leading to reduced Th1 and Th17 activation and attenuation of inflammatory processes, as shown in Figure 3 [11].

The high selectivity of deucravacitinib for TYK2 makes it especially valuable in treating immune-mediated disorders such as plaque psoriasis, as it lowers the likelihood of adverse effects often seen with broader JAK inhibition, including infections, blood abnormalities, and thromboembolic events. Evidence from clinical trials shows that deucravacitinib achieves strong efficacy in alleviating symptoms while offering a safety profile that is more favorable than traditional oral treatments for plaque psoriasis [3]. As research progresses, tailoring JAK inhibition to specific disease states and genetic profiles will shape the next generation of medicine. While these drugs continue to advance therapeutic boundaries, their long-term benefits and risks remain an important area of ongoing investigation.

Efficacy in Clinical Trials of Risankizumab - IL-23 Inhibitor

In phase III randomized clinical trials, risankizumab demonstrated greater efficacy than placebo at week 4, achieving higher PASI 90 response rates and lower relapse rates, with relapse defined as a Static Physician’s Global Assessment (sPGA) score of ≥3. When measuring efficacy following treatment withdrawal, participants who discontinued risankizumab and were later reintroduced at week 40 regained higher levels of disease control compared with those who maintained treatment withdrawal. Additionally, participants who missed their dose at week 28 showed lower efficacy than those with continued dosing; however, reintroduction of risankizumab restored efficacy within 16 weeks. These findings highlight the consistent therapeutic benefit of risankizumab when used on a continuous schedule. Long-term follow-up from phase III studies has shown sustained efficacy over two years with a standard 12-week dosing regimen for this IL-23 inhibitor [10].

Risankizumab also demonstrated robust reductions in plaque psoriasis severity with a 150 mg dose administered at four-week intervals over a 16-week period, as assessed by Person Specific Outcome (PsO) scores. These improvements were maintained through week 52 with comparable efficacy to earlier time points. In the five-year open-label extension study LIMMitless, risankizumab demonstrated durable disease control and maintained high PASI response rates, reinforcing its long-term efficacy in the management of plaque psoriasis [13].

Safety Profile of Risankizumab

In the UltlMMa-1 and UltlMMa-2 phase III trials, risankizumab demonstrated similar safety profiles when compared to placebo and ustekinumab in treating patients with plaque psoriasis. No unexpected safety signals emerged, even when patients switched from placebo to risankizumab [10]. In the LIMMitless long-term extension study, safety outcomes remained stable, with the most common adverse events consisting of nasopharyngitis, upper respiratory tract infection and arthralgia. Serious adverse events, events leading to discontinuation, and deaths were infrequent, with no deaths specifically attributed to risankizumab. Key safety concerns include cardiovascular events, cerebrovascular accidents, malignancies, and serious infections, which occurred at low rates in the psoriasis patient population. These results support a favorable long-term safety profile for the IL-23 inhibitor in the use of biologic plaque psoriasis treatment [13].

Efficacy in Clinical Trials of Bimekizumab - IL-17 Inhibitor

Following phase IIb and phase III clinical trials, bimekizumab demonstrated substantial reduction in plaque psoriasis severity, as measured by PASI 90 and PASI 100 scores, which directly correlate with DLQI. After pharmacokinetic (PK) and pharmacodynamic (PD) evaluation, bimekizumab maintained consistent plaque reduction with an initial dosing regimen of every four weeks (Q4W), followed by every eight weeks (Q8W) as measured at week 16. In contrast, dosing at every 12 weeks (Q12W) was associated with reduced efficacy, making the eight-week dosing interval the most effective maintenance schedule [9]. Three-year follow-up data showed that PASI 90 and PASI 100 scores, along with DLQI improvements, remained comparable to those achieved with risankizumab, with significant plaque reduction maintained on the Q8W maintenance schedule after the initial 16 weeks [14].

Safety Profile of Bimekizumab

In the phase IIa randomized, double-blind, multicenter study of bimekizumab in moderate-to-severe plaque psoriasis by Oliver et al., adverse events were more significant in bimekizumab-treated patients in comparison to placebo groups, however with mild to moderate severity. Commonly reported adverse events included nasopharyngitis, upper respiratory tract infections, and oral candidiasis occurring most frequently. No deaths or unexpected safety signals were observed, and serious adverse events were infrequent and balanced between treatment and placebo groups [9]. In the BE READY study, bimekizumab demonstrated a favorable safety profile, with treatment-emergent adverse events (TEAEs) reported in 61% of patients receiving bimekizumab 320 mg every four weeks compared with 41% of those on placebo. The most common events were the same as those seen in phase IIa studies of the IL-17 inhibitor [14].

Efficacy in Clinical Trials of Deucravacitinib - TYK2 JAK Inhibitor

Deucravacitinib has demonstrated durable clinical efficacy and an acceptable safety profile in both early-phase and pivotal trials [15]. In the phase III POETYK PSO-1 and POETYK PSO-2 studies, deucravacitinib significantly outperformed placebo and apremilast across multiple clinical outcomes, including PASI 75 and sPGA scores of 0 or 1 [15]. These trials also showed marked improvements in PROs, providing compelling evidence of deucravacitinib’s ability to improve both symptoms and overall quality of life for patients with psoriasis [16].

From a patient-centered perspective, deucravacitinib produced meaningful improvements in PROs, including the DLQI and the Psoriasis Symptom and Sign Diary (PSSD) [16]. Noticeable benefits appeared as early as one week after treatment initiation and were maintained through 52 weeks [17]. These early and sustained gains are particularly important when compared with traditional therapies and reflect the high proportion of patients reporting relief from itch, scaling, and flaking, symptoms that often disrupt daily life [16]. Findings from clinical efficacy trials reinforce these observations and highlight the value of incorporating PROs alongside standard clinical endpoints to better capture treatment effectiveness and patient satisfaction [15].

PK and PD data from early-phase studies further support the clinical utility of deucravacitinib. The medication is quickly absorbed after oral dosing and reaches steady-state levels within five days [6]. This rapid onset is important in psoriasis management, where timely symptom control can meaningfully improve patient quality of life. Deucravacitinib exhibits dose-proportional increases in peak plasma concentrations at lower doses, with consistent PK profiles observed even at higher doses, supporting the convenience of once-daily dosing [6]. The PK/PD relationship suggests that relatively low daily doses are sufficient to achieve sustained TYK2 inhibition, including IL-12-mediated interferon-gamma production and IFNα gene expression. These inhibitory effects are dose-dependent and achieved with clinically relevant plasma concentrations [6].

Safety Profile of Deucravacitinib

Deucravacitinib showed good tolerability in both single and multiple dosing regimens lasting up to 12 days during early-phase trials [6]. Skin reactions, such as mild rashes, occurred in a small percentage of participants, particularly at the higher dose of 12 mg twice daily, and were manageable with topical treatment. These findings were consistent with results from the POETYK PSO-1 and PSO-2 trials, where deucravacitinib demonstrated stable efficacy and a favorable safety record over four years in patients with moderate to severe plaque psoriasis [18]. Clinical improvements, including PASI 75 and sPGA 0 or 1, were maintained or enhanced over time, and exposure-adjusted adverse event rates remained steady without new safety concerns [18].

Combined efficacy and safety data from the phase III POETYK trials, along with supportive PK and PD findings, reinforce deucravacitinib as a strong systemic option for moderate to severe plaque psoriasis. Its rapid onset of effect, sustained benefits for up to 52 weeks, and consistent results across various patient subgroups highlight its broad potential in clinical practice.

Deucravacitinib’s success in psoriasis has prompted investigation into its potential use in other immune-mediated diseases. Its selective TYK2 inhibition has been shown to modulate immune responses, notably by attenuating IFNα-induced lymphocyte reduction, a hallmark of systemic lupus erythematosus (SLE). In SLE, IFNα promotes lymphocyte migration to lymphoid tissues, leading to peripheral lymphopenia and increased disease activity. By disrupting this pathway, deucravacitinib emerges as a compelling candidate for conditions where type I interferon signaling is implicated [6]. A summary of the findings is shown in Table 3. Only studies reporting extractable clinical efficacy and/or safety outcomes in patients with moderate-to-severe plaque psoriasis were included. Outcomes are reported as described in the original studies without cross-study comparison or interpretation.

**Table 3:** Summary of comparative efficacy trends and safety considerations for risankizumab, bimekizumab, and deucravacitinib. MAIC = matching-adjusted indirect comparison, PASI = Psoriasis Area and Severity Index, RCT = randomized controlled trial, sPGA = Static Physician’s Global Assessment

Study (Author, Year)	Intervention	Methodology	Results
Strober et al., 2023 [3]	Deucravacitinib	Phase III RCT	PASI 75/90; safety vs. placebo/apremilast
Oliver et al., 2022 [9]	Bimekizumab	Phase IIa RCT	PASI response; PK/PD; safety
Blauvelt et al., 2020 [10]	Risankizumab	Phase III RCT	PASI maintenance vs. withdrawal
Papp et al., 2023 [13]	Risankizumab	Open-label extension	Long-term PASI response; safety
Gordon et al., 2021 [14]	Bimekizumab	Phase III RCT	PASI 90/100; safety
Armstrong et al., 2024 [15]	Deucravacitinib	Phase III RCT	Patient-reported outcomes
Strober et al., 2024 [16]	Deucravacitinib	Pooled Phase III	Safety and tolerability
Korman et al., 2024 [17]	Deucravacitinib	Phase III RCT	Onset and maintenance of PASI response
Armstrong et al., 2025 [18]	Deucravacitinib	Long-term extension	4-year efficacy and safety
Gordon et al., 2018 [23]	Risankizumab	Phase III RCT	PASI 75/90/100 vs. placebo/ustekinumab
Armstrong et al., 2024 [24]	Risankizumab versus deucravacitinib	MAIC	Indirect comparative efficacy
Armstrong et al., 2023 [27]	Deucravacitinib versus adalimumab	MAIC	Long-term comparative efficacy

Discussion

Both IL-17 and IL-23 cytokine inhibitors, represented by bimekizumab and Risankizumab, respectively, have consistently demonstrated strong efficacy in reducing plaque psoriasis severity. The recently approved TYK2 inhibitor deucravacitinib shows comparable efficacy and safety outcomes, with the additional benefit of oral administration. A thorough comparison of these therapies that considers route of administration, speed of symptom relief, and long-term safety is essential for understanding potential patient benefits and informing therapeutic decisions in moderate to severe plaque psoriasis.

Patient convenience and comfort in self-administering treatment are important factors influencing adherence in chronic conditions that require long-term therapy. Incorporating patient tolerability into treatment decisions can also contribute to improved quality of life. In the FORTIFY on-body injector substudy, risankizumab was evaluated for real-world usability and patient experience in Crohn’s disease [19]. Most participants were able to successfully administer the full dose, and overall treatment satisfaction was high. A small number of participants experienced injection site reactions, including localized erythema or pain, and some reported mild discomfort associated with adhesive removal. Device misuse occurred in a minority of cases, resulting in premature drug release and incomplete dosing. Despite these events, risankizumab was generally well tolerated, and the findings highlight the importance of considering device handling, patient training, and user familiarity when selecting injectable biologic therapies [19].

There are no direct studies evaluating patient preference for TYK2 inhibitors compared with interleukin inhibitors in plaque psoriasis; however, data from other disease settings offer useful insight. In patients with myelodysplastic disorders, 75% expressed a preference for oral therapy over subcutaneous, intravenous, or other parenteral routes. Reported reasons included reduced interference with daily activities and greater convenience compared with injectable regimens [20]. These concerns may be even more relevant for elderly patients, who may have mobility limitations, transportation challenges, or discomfort with self-injection, all of which can contribute to decreased adherence. Oral medications such as deucravacitinib may provide a more accessible and less invasive alternative, promoting consistent and sustained disease control. In chronic conditions such as plaque psoriasis that require long-term therapy, treatment convenience, particularly the mode of administration, plays a critical role in adherence and patient comfort. A US survey performed in 2024 found that over 65 percent of patients prefer oral therapies because they induce less anxiety compared to injections or infusions [21]. An international preference study further showed that when efficacy and safety are held constant, more than 80 percent of both patients and dermatologists favored once daily oral treatments over biweekly injections [22]. The combination of patient-centered delivery, effective symptom control, and a favorable tolerability profile positions TYK2 inhibitors as an important advancement in psoriasis care. Further research is warranted to directly assess patient preferences and real-world adherence when comparing oral TYK2 inhibitors with biologic interleukin inhibitors, particularly as more oral immunomodulators become available for the treatment of psoriasis.

The speed of onset is an important consideration for patients, particularly for those whose quality of life is significantly impaired by psoriasis. In the phase III UltIMMa-1 and UltIMMa-2 trials, risankizumab achieved high efficacy in patients with moderate to severe plaque psoriasis, with up to 88% reaching PASI 90 at week 16 compared with 5% on placebo and 42% on ustekinumab [23]. Bimekizumab has demonstrated an even faster onset. PASI 90 was achieved in some patients by week 8, and PASI 100 peaked at week 12 before the second dose. Transcriptomic analyses at week 8 revealed near-complete normalization of psoriatic gene expression, including suppression of IL-17A, IL-17F, and IL-23 [9].

In the phase III POETYK PSO-1 and PSO-2 trials, deucravacitinib produced measurable PASI improvement as early as week 1 [17]. By week 16, substantial proportions of patients achieved PASI 75 and PASI 90, and these responses were maintained through week 52, demonstrating durable one-year effectiveness [18]. Indirect comparisons suggest that risankizumab achieves higher PASI 100 rates than deucravacitinib at week 16, indicating more complete skin clearance in some populations [24]. Although efficacy outcomes differ, deucravacitinib’s PK and PD profile as a selective TYK2 inhibitor provides the advantage of oral administration with the potential for long-term safety. Data are still limited due to its recent approval, but evidence from other JAK inhibitors in dermatologic and autoimmune diseases indicates stable tolerability over extended use. In a five-year study of patients who switched from adalibinumab (a biologic injectable) to upadacitinib (a JAK inhibitor) for rheumatoid arthritis, no new safety concerns emerged and the overall profile was consistent with earlier findings [25]. Adverse events that occurred more frequently included herpes zoster infection, lymphopenia, hepatic disorders, elevated creatine phosphokinase, and non-melanoma skin cancer, but these did not alter the overall favorable benefit-risk balance [25]. Additional evidence from an open-label follow-up study evaluating upadacitinib (a JAK inhibitor) in patients with atopic dermatitis who had previously received dupilumab (a biologic injectable) showed a consistent safety profile without any new concerns [26].

Indirect comparative analyses suggest that deucravacitinib may provide greater long-term efficacy than adalimumab in moderate-to-severe plaque psoriasis. At week 112, adjusted analyses demonstrated higher PASI 75 response rates with deucravacitinib compared with adalimumab, along with numerically greater PASI 90 responses [27]. These findings indicate that beyond the convenience of oral administration and a favorable safety profile, deucravacitinib may also deliver superior skin clearance and improved PROs, supporting its potential as a preferred systemic oral option for certain patients. The summary of these key findings is presented in Table 4.

**Table 4:** Summary of key findings discussed in the discussion section. IL = interleukin, JAK = Janus kinase, PASI = Psoriasis Area and Severity Index

Study Topic	Population/Disease Area	Intervention	Comparator	Key Efficacy Findings
FORTIFY on-body injector substudy for risankizumab [19]	Crohn’s disease	Risankizumab via on-body injector	None (usability assessment)	High rate of successful full-dose self-administration. High patient satisfaction.
Patient preference study in myelodysplastic disorders [20]	Myelodysplastic syndromes	Oral therapy	Subcutaneous, intravenous, or parenteral agents	75% preferred oral therapy due to convenience and less interference with daily activities.
United States 2024 survey on treatment preference [21]	Adults with chronic diseases requiring long-term therapy	Oral therapies	Injections or infusions	Over 65% preferred oral treatments due to reduced anxiety and convenience.
International patient and dermatologist preference study [22]	Plaque psoriasis patients and dermatologists	Once daily oral medication	Biweekly injections	More than 80% preferred daily oral therapy when efficacy and safety were equivalent.
Risankizumab UltIMMa-1 and UltIMMa-2 [23]	Moderate to severe plaque psoriasis	Risankizumab	Placebo and ustekinumab	Up to 88% percent achieved PASI ninety in week 16 compared with 5% on placebo and 412% on ustekinumab.
Bimekizumab transcriptomic/onset data [9]	Moderate to severe plaque psoriasis	Bimekizumab	None or active comparator depending on trial	PASI 90 achieved by week 8 in some patients. PASI 100 peaked at week 12. Week 8 transcriptomics showed near-complete normalization of psoriatic gene expression including IL-17A, IL-17F, and IL-23 suppression.
Deucravacitinib POETYK PSO-1 and PSO-2 [17,18]	Moderate to severe plaque psoriasis	Deucravacitinib	Placebo or apremilast	Measurable PASI improvement as early as week 1. By week 16 substantial PASI seventy-five and PASI ninety responses achieved, maintained through week 52.
Risankizumab versus deucravacitinib Indirect comparison [24]	Moderate to severe plaque psoriasis	Risankizumab or deucravacitinib (indirect)	Indirect comparison	At week 16 risankizumab achieved higher PASI 100 rates than deucravacitinib.
Upadacitinib five-year rheumatoid arthritis extension study (JAK inhibitor safety context) [25]	Rheumatoid arthritis patients switching from adalimumab	Upadacitinib	Prior adalimumab	Not discussed for efficacy in psoriasis. Provides supportive long-term JAK inhibitor safety.
Upadacitinib open-label follow-up in atopic dermatitis [26]	Atopic dermatitis patients previously treated with dupilumab	Upadacitinib	None	Not efficacy focused on discussion. Supports generalizability of JAK inhibitor safety.
Deucravacitinib versus adalimumab long-term indirect comparison [27]	Moderate to severe plaque psoriasis	Deucravacitinib	Adalimumab	Higher PASI 75 and numerically higher PASI 90 responses at week 112 with deucravacitinib. Suggests superior long-term skin clearance.

## Conclusions

IL-17 and IL-23 inhibitors, bimekizumab and Risankizumab, remain highly effective options for moderate to severe plaque psoriasis, while deucravacitinib emerges as a promising oral alternative with a unique mechanism and encouraging efficacy and safety data. Its oral route of administration offers a significant advantage in terms of convenience and patient adherence when compared to injectable biologics. The early and sustained PASI responses observed with deucravacitinib, beginning as early as week 1, are particularly valuable for patients seeking fast relief from the burdensome symptoms of plaque psoriasis. While long-term data are still being collected, current findings suggest that deucravacitinib maintains efficacy and is associated with fewer severe adverse events than those typically reported with earlier JAK inhibitors and some biologics. With its targeted TYK2 inhibition, which provides reduced systemic immunosuppression and a lower risk of serious infections or laboratory abnormalities, deucravacitinib holds strong potential to become a first-line treatment option for moderate to severe plaque psoriasis, offering both efficacy and convenience without compromising safety. Nevertheless, further long-term and comparative studies are needed to better define its durability, safety, and optimal place in psoriasis treatment algorithms.

## Appendices

**Table 5:** Comprehensive database search log

Database	Search Number	Date Run	Search Terms	Records Retrieved (n)	Screening Approach/Notes
PubMed	1	01/20/2025	‘Risankizumab AND plaque psoriasis’	212	Full text available
PubMed	2	01/21/2025	‘Deucravacitinib AND plaque psoriasis'	20	English, 5 years, free full text
PubMed	3	01/21/2025	‘Sotyktu’	9	English, 5 years, free full text
PubMed	4	01/21/2025	‘Deucravacitinib AND JAK inhibitors'	51	English, 5 years, free full text
PubMed	5	01/21/2025	‘JAK inhibitors AND interleukin inhibitors AND plaque psoriasis’	2	English, 5 years
PubMed	6	01/23/2025	‘Bimekizumab AND plaque psoriasis'	95	Full text available
PubMed	7	01/26/2025	‘Ixekizumab AND plaque psoriasis’	372	Full text available
PubMed	8	01/26/2025	‘Deucravacitinib AND risankizumab’	13	None
PubMed	9	01/26/2025	‘Deucravacitinib AND bimekizumab’	8	None
PubMed	10	01/26/2025	‘Deucravacitinib AND ixekizumab’	8	None
PubMed	11	01/29/2025	‘Risankizumab AND JAK inhibitors'	22	Full text, 5 years
PubMed	12	01/29/2025	‘Bimekizumab AND JAK inhibitors'	8	Full text, 5 years
PubMed	13	01/29/2025	‘Ixekizumab AND JAK inhibitors'	14	Full text, 5 years
PubMed	14	01/29/2025	‘Interleukin inhibitors AND JAK inhibitors AND plaque psoriasis’	14	5 years
PubMed	15	01/29/2025	‘Interleukin inhibitors AND plaque psoriasis’	70	5 years, free full text
PubMed	16	01/29/2025	‘Bimekizumab AND plaque psoriasis (article type specified)’	21	English, 5 years, free full text
PubMed	17	03/09/2025	‘Ustekinumab (Stelara) AND plaque psoriasis’	506	Full text
PubMed	18	03/09/2025	‘Plaque psoriasis treatments’	6,239	None
Embase	19	03/09/2025	‘Plaque psoriasis AND risankizumab'	561	English, human studies
Embase	20	03/09/2025	‘Plaque psoriasis AND bimekizumab’	308	English, human studies
Embase	21	03/09/2025	‘Plaque psoriasis AND deucravacitinib'	266	English, human studies
Embase	22	03/09/2025	‘Plaque psoriasis AND sotyktu’	16	English, human studies
Embase	23	03/09/2025	‘Plaque psoriasis AND interleukin inhibitor’	3	English, human studies
Google Scholar	24	03/09/2025	‘"Plaque psoriasis" risankizumab’	5,490	First 200 results screened by relevance
Google Scholar	25	03/09/2025	‘"Plaque psoriasis" bimekizumab’	2,730	First 200 results screened by relevance
Google Scholar	26	03/09/2025	‘Plaque psoriasis deucravacitinib’	2,730	First 200 results screened by relevance
Google Scholar	27	03/09/2025	‘Plaque psoriasis sotyktu’	316	First 200 results screened by relevance
Google Scholar	28	03/09/2025	‘Plaque psoriasis interleukin inhibitor’	23,500	First 200 results screened by relevance
